# Current process and future path for health economic assessment of pharmaceuticals in France

**DOI:** 10.3402/jmahp.v3.27902

**Published:** 2015-06-04

**Authors:** Mondher Toumi, Cécile Rémuzat, Emna El Hammi, Aurélie Millier, Samuel Aballéa, Christos Chouaid, Bruno Falissard

**Affiliations:** 1 Faculté de Médecine, Laboratoire de Santé Publique, Aix-Marseille Université, Université de la Méditerranée, Marseille Cedex, France; 2 Creativ-Ceutical, Paris, France; 3 Evidenz, Tunis, Tunisia; 4 INSERM U955 and Université Paris Est (UPEC), UMR U955, Faculté de médecine, F-94010, Créteil, France; 5 Centre Hospitalier Intercommunal, DHU-ATVB, Département de Pneumologie et Pathologie Professionnelle, F-94000, Créteil, France; 6 INSERM Unit U669 (Public Health and Mental Health), University Paris-Sud, Maison de Solenn, Paris, France

**Keywords:** health economic assessment, CEESP, HAS, France, market access, cost-effectiveness

## Abstract

The Social Security Funding Law for 2012 introduced the Economic and Public Health Assessment Committee (Commission Evaluation Economique et de Santé Publique, or CEESP) in the Social Security Code as a specialised committee affiliated with the Haute Autorité de Santé in charge of providing recommendations and health economic opinions. This article provides an in-depth description of the CEESP's structure and working methods, and analyses the impact of health economic assessment on market access of drugs in France. It also points out the areas of uncertainty and the conflicting rules following the introduction of the health economic assessment in France. The authors also provide their personal opinion on the likely future of health economic assessment of drugs in France, including the possible merge of the CEESP and the Transparency Committee, the implementation of a French threshold, and the extension of health economic assessment to a larger number of products.

With the introduction of the Social Security Funding Law for the year 2008, the French National Authority for Health (Haute Autorité de Santé, or HAS) has been commissioned to produce ‘health economic opinions’, determine the most cost-effective therapeutic strategies, and edit the recommendations accordingly (1). An affiliate organ named the Economic and Public Health Assessment Committee (Commission Evaluation Economique et de Santé Publique, or CEESP) has been set up to fulfil this mission. The Social Security Funding Law for 2012 (2) introduced the CEESP in the Social Security Code as a specialised committee in charge of providing recommendations and health economic opinions. This decision came as part of an effort not only to confront the increasing deficit of the Social Security System and encourage it to recover a healthy financial balance but also in response to the French Court of Auditors (Cours des Comptes), which regularly challenged the lack of economic evidence use in pricing decisions (3).

The law and the application decree still leave areas of uncertainty, making it difficult to appreciate the place and role of health economics. There are evident conflicting rules following the introduction of the health economic assessment in France that request clarification and resolution. The current situation should be considered to be a transitional period, and major steps are expected in the near future.

This article aims to provide an in-depth description of the CEESP's structure and working methods, as well as to analyse the impact of health economic assessment on market access of drugs in France. It may help readers appreciate the current management of conflicting situations and put it in context in the overall health technology assessment (HTA) in France. It also provides likely directions of future French HTA organisation and processes.

## Description of the CEESP

### Context of the CEESP implementation and legislation

It was the Social Security Funding Law for 2008 that first assigned the HAS the task of producing recommendations and health economic opinions on the most cost-effective medical care and prescription strategies (Article L161-37) (1).

The CEESP was created for this purpose in 2008, 4 years after the creation of the HAS. However, up until 2012, this committee operated as an internal group within HAS and exclusively provided health strategic advice. Until then, it was not mentioned in the Social Security Code and did not exist as an independent legal entity within HAS. Therefore, the opinions and recommendations issued by the CEESP had a relatively low impact on the pricing and reimbursement of health products. Moreover, the CEESP used to review old products with limited question marks on pricing (4).

**Fig. 1:**
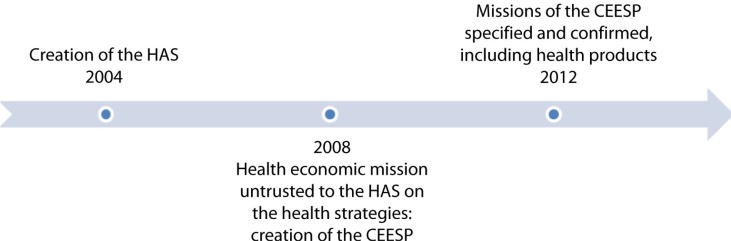
Establishment of the CEESP.

In an effort to enhance the financial sustainability of the healthcare system, the Social Security Funding Law for 2012 introduced the CEESP as a specialised committee under Article 47 of the Social Security Code, in charge of providing recommendations and health economic opinions. Concurrently, the board and structure of this committee were remodelled to suit these new prerogatives (Fig. 1) (2).

### Aim of the CEESP

The establishment of the CEESP is part of an effort to ascertain that both public and professionals’ decisions in the medical field (particularly in terms of pricing and reimbursement) take into consideration the notions of cost-effectiveness and opportunity cost.

Within the framework of its core mission, the CEESP is expected to guarantee the scientific validity, the methodology, and the ethics of the work that HAS conducts in economic and public health evaluation (5). Topics with a high potential for expense optimisation are prioritised, particularly during the reassessment of therapeutic classes or the assessment of medical care strategies from a medium-term perspective (5).

### Missions

According to the CEESP rules of procedure (6) and within the framework of the missions entrusted to it, the committee is expected to deliver health economic opinions on medical procedures, products, or health services (Article L.161-37, 1st paragraph of the Social Security Code); to perform or validate the health economic studies necessary for the evaluation of health products and technologies (Article L.161-37, 1st paragraph of the Social Security Code); and to set up or spread health economic recommendations on the most cost-effective care, prescription, or coverage strategies.

**Fig. 2:**
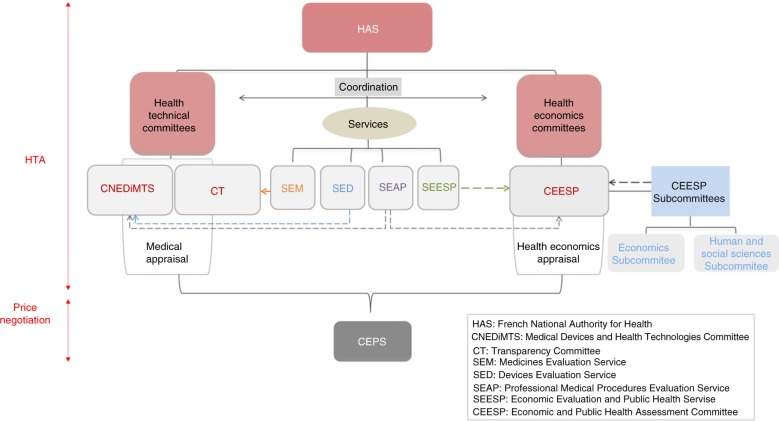
Organisation of the French HTA and pricing system. Dashed arrows indicate the support of the subcommittees/services to the different committees in their work.

**Fig. 3:**
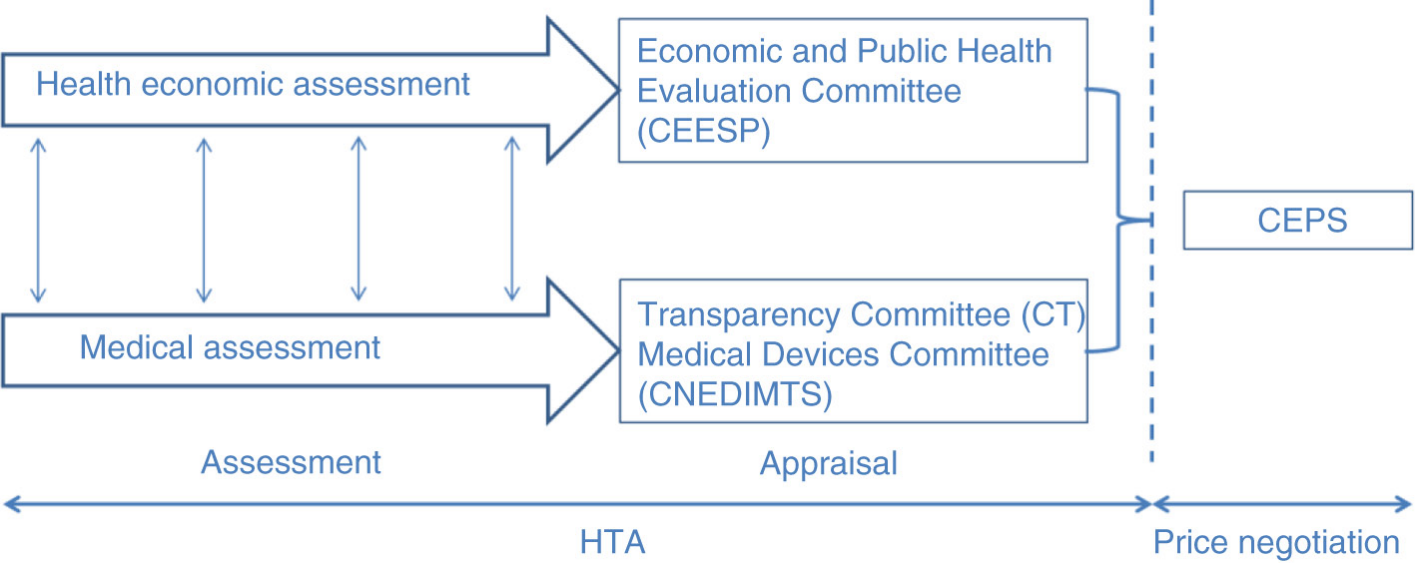
The coordinated assessment/appraisal (8).

The CEESP relies on the works of two services: the Service of Economic Evaluation and Public Health (Service évaluation économique et de santé publique, or SEESP) and the Service of Evaluation of the Professional Medical Procedures (Service Evaluation des Actes Professionnels, or SEAP) (5) (Fig. 2). It is also required to coordinate its health economic appraisal with the medical appraisal performed by the Transparency Committee (Commission de la Transparence, or CT) and the Medical Devices and Health Technologies Committee (Commission Nationale d'Evaluation des Dispositifs Médicaux et des Technologies de Santé, or CNEDIMTS) (5) (Fig. 3). The Device Evaluation Service (Service Evaluation des Dispositifs Médicaux, or SED) and the Medicines Evaluation Service (Service Evaluation des Médicaments, or SEM) are in charge of examining the dossiers for the CNEDIMTS and the CT, respectively. All of the committees form part of the HAS and deliver their opinions to the Economic Committee on Healthcare Products (Comité Economique des Produits de Santé, or CEPS), which is under the joint authority of the ministers in charge of Health, Social Security, and Economy. The CEPS is in charge of setting the prices of medicinal products for individual use, which are covered by the national health insurance (7) (Fig. 2).

Upon the request of the HAS College, the CEESP can also be instructed to deliver its deliberations concerning:

- The opinions referred to in Article L.161-40 of the Social Security Code on the list of periodic preventive medical consultations and the screenings conducted as part of the health programs referred to in Article L.161-37 of the Social Security Code
- Assessments of the public healthcare quality in accordance to Article L.161-40, 3rd paragraph of the Social Security Code
- The opinions related to the medical procedures with aesthetic aims (Article L. 1151-3 of the Social Security Code)
- The works that can be useful for the accomplishment of HAS’ missions within the competence field of the CEESP (6).

### Composition

The CEESP is composed of 33 members with voting rights, appointed by the College of the HAS for a period of 3 years, which is renewable twice. The members include the president (appointed from the College members), health professionals, personalities appointed for their expertise in economic evaluation and public health fields, and representatives of users’ or patients’ associations. Two vice presidents are elected by the College among these members.

In addition to the permanent members, other persons who can play an advisory role can attend the committee meetings like the representatives of ministers in charge of Health and Social Security, representatives of health insurance organisations, as well as other individuals from within or outside the HAS. Any member of the College as well as the director can attend the committee meetings.

The CEESP committee is composed of one-third economists, one-third health professionals (including public health doctors, epidemiologists, and field doctors), as well as one-third representatives of social and human sciences. This composition reflects the objective of the CEESP: to combine the economic evaluation and public health aspects (6).

### Functioning

The secretariat is provided by the SEESP. It offers the administrative help necessary for the functioning of the CEESP and coordinates the works of the latter with the activities of the other HAS committees.

In order to prepare its work, the CEESP meets in subcommittees. More specifically, an economics subcommittee and human and social science subcommittee can be convened to proceed to prior methodological analyses (Fig. 2). The subcommittees are chaired by a member of the committee, and the minutes of the meeting are then transmitted to the committee.

The bureau of the CEESP is composed of the president, the two vice presidents, the two presidents of the subcommittees, as well as the department head of the SEESP. The missions of the bureau are to prepare the meetings of the committee, to set the agenda, to appoint the rapporteurs among the members of the committee, to examine the potential conflicts of interest of the external experts (and validate their participation), as well as to rule on the written observations presented by the companies during the adversarial phase, when these observations are related to the form and not the content of the opinions.

The periodicity of the CEESP meetings depends on the number of files the committee has to examine. The agenda and the documents related to the agenda items as well as a draft of the record of the previous meeting are filed to each member of the committee at least 5 days before the committee meeting.

The rapporteurs are in charge of inspecting the relevance and feasibility of the proposed works as well as their methodological quality in accordance with the predefined methods. They deliver their written report before the meeting.

The president of the committee determines the schedule, and convenes and chairs the meetings. If he or she is absent or unable to act, the presidency of the meeting is entrusted to a vice president or to another member of the committee.

The committee can only deliberate if a majority of its members are present. If this quorum is not met, the committee must postpone the session. At the second meeting, however, they must deliberate, no matter how many members are present. The results of the voting are established by a simple majority of the present members.

The meetings of the committee are recorded, in accordance with Article L.1451-1-1 of the Social Security Code. The recordings are retained by the services of the HAS and can be published on the website of the HAS, upon the request of the president of the HAS.

The minutes of the meetings are made public and contain the agenda and the report of the meeting. The latter includes the date of the meeting, the list of the members present and those excused, the topics examined, the participation and non-participation of the members of the committee in view of the possible links of interest, the content of the debates, the results of the voting, and their possible explanation. The minutes are submitted for approval to the committee during the following session. They are then circulated to the members of the committee, all the participants of the meeting, the director of the HAS, the members of the College, and all of the representatives of the ministers in charge of Health and Social Security. They are retained and archived by the secretariat of the committee and published on the HAS website.

Every year, the committee elaborates an activity report that is presented to the Parliament according to Article L. 161-37 of the Social Security Code. This report includes information related to the health economic opinions rendered during the year in question and the guidelines of the committee defined during the examination of the files (6).

## Health economic evaluation

### Scope: for which products?

Decree No. 2012-1116 of 2 October 2012, related to the health economic missions of the HAS, specifies the cases for which a health economic assessment will be required for drugs and medical devices (9).

In accordance with this decree, two criteria must be met in order to proceed to the health economic assessment of a health product:

1. The Improvement of the Medical Benefit (Amélioration du Service Médical Rendu, or ASMR) or the improvement of the benefit (Amélioration du Service Attendu, or ASA) claimed by the company is major, important, or moderate (ASMR or ASA I, II, or III); and
2. The health product is susceptible to having a significant impact on the health insuranprofessional practices, or patient care and, when applicable, its price.

In 18 September 2013, the HAS complemented the above definition of the range of products subject to health economic assessment by setting a threshold of €20 million yearly revenue to define the significant impact on the health insurance budget. Therefore, a health economic evaluation is now also required for first listing and relisting of drugs and medical devices with yearly projected revenues of €20 million or above.

The HAS College considers that economic valuation is not required:

1. If a conventional price drop procedure is initiated; or
2. If the product patent has expired (10).

### General rules and guiding principles

In accordance with Decree No. 2012-1116 of 2 October 2012, health economic evaluation is conducted by the CEESP, concomitantly and independently of the CT assessment, and following a guideline related to methodological choices for economic assessment issued by the HAS in 2011 (11).

The guideline is very flexible even if some specific recommendations were made (e.g., no cost–benefit analysis, loss of productivity not included in reference case, and deterministic and probabilistic sensitivity analysis required). With regard to the methodology of evaluation, the committee remains open to all options, as long as they are well argued and scientifically sound (11).

The burden of proof lies with the manufacturer, who must produce the evidence that supports its claim in terms of ASMR/ASA, projected revenues and impact on healthcare organisations, professional practices, and patient care. Data are submitted by the manufacturer to both the CEESP and the CEPS, along with the request for inclusion/renewal of inclusion of the product on the reimbursable drugs formulary. The submitted evidence is scrutinised by the HAS board to determine the product's eligibility for health economic evaluation (5, 6).

### File composition

The pharmaceutical company, along with the request for inclusion/renewal of inclusion of a medicine on the reimbursable drugs formulary, must transmit all health economic data related to the drug to the CEESP and to the CEPS, if the product falls under any of the categories concerned with health economic assessment. This was detailed in the ‘Scope’ section of this article (12).

The composition of the file must be as follows:

1. A *depositing slip* available for download from the HAS website and labelled ‘Depositing slip for a file to be examined by the Economic and Public Health Assessment Committee’. This form must be filled out by applicants and will be returned to them as an acknowledgement of receipt of their file.
2. A *presentation report* written in French containing all the necessary data for the health economic evaluation. A template of the presentation report is available for download from the HAS website (13). It must contain the following information:
  - General information about the application.
  - Summary presentation of the cost-effectiveness evaluation.
  - Objectives and results of the submitted study.
  - Methodological choices providing the structure for the cost-effectiveness evaluation.
  - Checklist labelled ‘Methods of evaluation of budget impact’ (budget impact is not mandatory).
  - Checklist labelled ‘Methods of evaluation of cost-effectiveness’.
  - Inventory of existing cost-effectiveness studies.
  All files must follow this standard layout, and the information contained in the presentation report must be consistent with the content of the technical reports.
3. *Technical reports* can be drafted in French or English. However, the choice of language must be uniform for all of the technical reports produced. For technical reports written in English, a French–English glossary of the technical terms is required. If a budget impact study has been conducted, it must be introduced in a separate technical report.
  The technical reports must allow the SEESP to appreciate the compliance of the working methods with the HAS guidelines, and they must describe the obtained results clearly and without ambiguity.
  The opinion delivered by the HAS is founded on a critical analysis of the study's conformity with the guidelines, hence the need for these reports to be extremely thorough and the chosen methodology well argued.
  The general objectives of the technical reports are outlined in the HAS procedural guideline as follows:
  - Present the context and the objectives of the analysis.
  - Present the retained methodological choices and data sources.
  - Explain and justify any dispensation or failure to meet the HAS guidelines and recommendations.
  - Present the results of the main analysis and those of the auxiliary sensitivity analysis.
  - Discuss the results with regard to the uncertainty levels and the results of other documented evaluations.
  - Present any complementary analysis conducted by the manufacturer.
4. *Computer files*
  The computer files containing the economic model and the budget impact study, when appropriate, must be provided to the CEESP.
  The committee favours the following software: Excel, Treeage, Treeplan, and poptools (Excel environment). The use of any other software must be justified, based on technical arguments. Furthermore, the necessary documentation for understanding of the models under a different software environment must be presented.
  The files must not be presented in read-only mode. The parameters must be clearly identified, and the values attached to them must be open for modification in order to allow unrestricted use of the model to appreciate the model dynamic and levels of uncertainty.
5. *Appendix*
  The following items must be attached to the file's appendix:
  - A copy of the file deposited at the CT or the CNEDIMTS.
  - A copy of the file deposited at the CEPS.
  - A copy of the bibliographic references of the file and, in particular, the scientific publications and the evaluation reports from other agencies.

## Timeline and steps of the procedure

### Early advice meetings

At the early stages of the health economic evaluation, and before conducting any studies on the cost-effectiveness of their product, the drug manufacturer has the option of consulting with the SEESP to discuss their methodological choices.

This procedure is referred to as ‘Early Meetings’, and it aims to ascertain that the health economic assessment work conducted by the manufacturer and submitted to the CEESP follows the methodological guidelines of the latter.

Furthermore, the early meetings allow the drug manufacturer to grasp the specific features of the disease, in terms of funding and coverage, as well as to conduct an overview of the available data and explore the relevant methods to complete it (14).

Early meetings can be conducted during, or before, the late-stage clinical trials. At this point, they aim to help the manufacturer design their trials in a way that allows them to collect the relevant data for the upcoming economic evaluation.

Likewise, the drug manufacturer is free to approach the SEESP at a later stage, when the clinical trials have been conducted successfully, in order to discuss the most relevant methodology for their health economic assessment.

This procedure is optional, nonbinding, confidential, and free of charge.

### Early advice application

When applying for this procedure, the drug manufacturer must file a dossier containing:

1. An application form.
2. A general description of the assessed product and the addressed pathology.
3. A detailed description of the economic evaluation protocol.
4. A list of questions related to methodological issues, addressed to the SEESP, as well as the company position on these questions.
5. The publications related to the evaluation model (only in case the evaluation protocol rests on a pre-existing model).

The HAS examines the dossier and checks whether there are sufficient grounds to organise an early meeting. If so, then the date is determined according to the available time slot.

### Early advice meeting

During the meeting, the drug manufacturer can be assisted by the authors of the study if they belong to a third-party organisation. However, the HAS guidelines state that the number of participants on behalf of the manufacturer must be ‘reasonable’.

The HAS personnel and representatives, as well as their appointed third-party experts, are all bound by a confidentiality clause and have no authority to release any of the information brought to their attention during the deliberations. However, the HAS will decline any proposition to sign a supplemental confidentiality agreement submitted by the manufacturer.

The experts appointed by the HAS for the purpose of these meetings cannot be hired by the manufacturer for the conduct of the study.

### Early advice meeting minutes

**Fig. 4:**
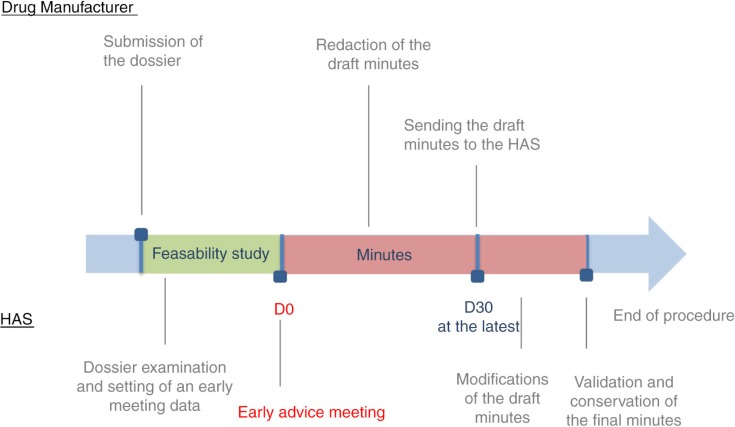
Steps of the early advice meeting.

After the meeting, the manufacturer is required to draft a meeting report following a simple structure that is outlined in the HAS guidelines. The report must be submitted to the HAS within 30 days of the end of the meetings. The report will be altered by the HAS representatives if necessary, and a copy will be returned to the manufacturer. The content of this report will not be published by the HAS (Fig. 4).

### Standard advice

The file submission by the manufacturer marks the official launch of the health economic appraisal procedure. The instruction time will be calculated starting from the day of receipt of the applicant's file by the CEPS.

**Fig. 5:**
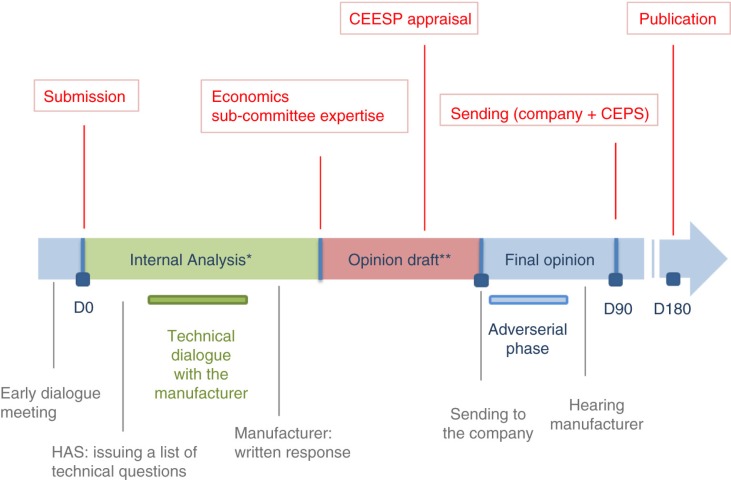
Economic appraisal process. Adapted from HAS, Economic appraisal process (8). *Administrative compliance and scientific/methodological compliance. Key actors: 2–3 project managers from the Health Economics and Public Health Department (SEESP) + economics sub-committee rapporteur + possibility to submit questions to external clinical and/or methodological experts. **Key actors: the CEESP members: economic, clinical public health, and social science experts (monthly meeting).

During the allocated period of 180 days, the file will go through the following steps (Fig. 5) (15).

#### Preliminary analysis

Immediately after receipt of the file by the HAS, the content will be checked for compliance with the administrative rules and guidance. In the event of any required documentation or information being missing, the instruction will be suspended until the completion of the file, and the processing times and deadlines will vary accordingly.

After validation by the administrative services, the file will be checked for admissibility of the study protocol. At this stage, the details of the protocol are not examined yet, but are overviewed to make sure the content of the file is relevant to determining the cost-effectiveness of the product. Otherwise, the file will be immediately returned to the applicant along with a statement of rejection.

#### Methodological analysis

This phase is conducted by the project managers of the SEESP. It is a standardised procedure conducted in accordance with the guiding principles stated in two reference documents issued by the HAS and the College of Health Economists (11, 16).

The critical analysis of the methodology is thoroughly discussed by the project managers of the SEESP with their counterparts of the SEM and SED. It is then drafted in detail. For each part of the study, the draft shows a neutral presentation of the methodology adopted by the authors of the study, followed by a thorough critical analysis. The draft is appended to the opinion form and will serve as a basis for further discussion by the economics subcommittee and the CEESP.

The dossier is then discussed during a meeting with the members of the economics sub-committee of the CEESP, and both parties agree on a list of questions concerning the methodology to be addressed to the manufacturer.

#### Questions to the manufacturer

After meeting with the members of the economics subcommittee, the project managers of the SEESP direct their questions to the manufacturer. The manufacturer has the option to either return written answers by the deadline, which is set to 8 days before the next meeting of the sub-commission, or apply to be present at the next meeting of the sub-commission within 8 days from receipt of the questions.

#### First draft

At their next meeting, the economics subcommittee examines the written or verbal answers of the manufacturer. A first opinion is then drafted in accordance with the initial methodology analysis and the manufacturer's answers. It is discussed and approved by the CEESP at their next meeting, and then forwarded to the manufacturer within 5 days.

#### Adversarial phase

Within 8 days of their receipt of the opinion draft, the manufacturer can either address a list of written observations or request an audience with the president of the CEESP. The audience is scheduled for the next meeting of the CEESP, or the one after in case of a short notice.

#### Final draft and opinion

The opinion draft can be altered after the audience with the manufacturer. In any event, a final opinion is adopted by the CEESP at the end of the hearing stage. It is then forwarded to the CEPS and to the manufacturer, and it is published on the HAS website after price negotiation.

## Discussion

### French specificities

There are three French specificities:

- The parallel process for the TC and the CEESP.
- Health economics is used for price setting and not reimbursement.
- The perspective of health economics evaluation is neither the health insurance nor the social perspective.

### Health economics encounters a big misunderstanding

Most reports supported that health economics should be introduced as a rationing tool to address budget constraints. In fact, health economics aims to support decision making and not to ration access. However, this confusion is not specific to France.

### Health economics has become an irrevocable part of the HTA process

In light of these changes, it is evident that cost-effectiveness studies will now be part of market access requirements for all drugs satisfying the selection criteria for health economic assessment (17). From 3 October 2013 to December 2014, 68 dossiers were examined by the CEESP, 26 of them were judged eligible (25 drugs and 1 medical device), and 11 dossiers were being examined at that date (18).

The application decree targets the budget impact, as well as the incremental benefit, as being the drivers for eligibility for health economics assessment of drugs. It has become apparent that the legislator's question was: ‘What should the cost be for a given added benefit?’ It is expected health economics will help addressing that question; however, the lack of clear reference cases and the lack of an Incremental Cost-Effectiveness Ratio (ICER) threshold will make it difficult to provide an answer. The CEESP is expected to inform on the compliance of health economic evaluations with the HAS guidelines, but not to inform on whether the intervention is cost-effective or not. The parliament debate and the new law impact study refer to *quality of life* as the effectiveness outcome for health economic assessment. This leaves little doubt on the expected preeminent role of quality-related life-years (QALYs), although this term was not pronounced or written in publicly available documents during the parliament debates. This is consistent with the CEESP-HAS guidelines.

### Major sources of inefficiencies should be addressed

The legislator in charge of the law setting and the executive administration in charge of issuing the application decree may have underestimated the source of inefficiencies associated with this new law.

- The number of cases for which a health economic assessment would be performed but not used for decision making was not taken into consideration in the drugs selection criteria for health economic assessment. Indeed, companies claim ASMR I, II, or III in a large proportion of cases, while only a few get such an outcome. For example, in 2013, for a first listing drug assessment, 1 ASMR I, 0 ASMR II, and 8 ASMR III were granted for 10 ASMR IV and 148 ASMR V (19, 20). Products that get ASMR IV or V are not expected to have an impact on the pharmaceutical budget according to French regulation. This is one of the important French pharmaceutical pricing rules (21). As such, if a product has claimed an ASMR I, II, or III but is eventually granted an ASMR IV or V by the CT, the health economic assessment that was already completed and submitted in theory should not be used for pricing decision making (17). As most of the submitted dossiers benefit from a consultation with the SEESP, the current process leads to a substantial waste of resources to manage and review those applications that will ultimately receive an opinion that is not supposed to be used. However, the CEPS claims to use it, but it is unclear how this impacts the price and rebate.
- There is a duplication of work within the same organisation, HAS, as both committees (the CEESP and the CT) assess the public health impact for any submitted intervention and, to a lesser extent, the effectiveness or even the efficacy. This may be considered another source of inefficiency.
- As both committees operate independently and concomitantly, it is urgent that a clear process is established to clarify the resolution of divergent opinions. While the CT only focusses on clinical evidence, the CEESP is expected to have a broader perspective but be driven initially by the same clinical evidence. Divergent assessment of the same evidence is possible or even likely, and it should be prevented for the consistency of overall HAS opinions. The chairman of HAS expressed publicly that such divergences will be resolved within the High College of HAS; however, no information on the process is available. It may not be the most appropriate place to address those divergences as the chairman of the College is also the chairman of the CEESP. Not only does that situation raise an issue about a transparent process of resolution of divergences, but such effort appears as another source of inefficiency.

### There are multiple sources of confusion

This decree creates confusion and conflicting information with respect to the current regulation and practices. It is can be observed that many of the HAS recommendations on the eligibility criteria for the health economic assessment of drugs were not implemented in the decree (20, 22).

- This new situation appears to conflict with regulation in force regarding the external reference pricing (ERP) (versus the United Kingdom, Germany, Spain, and Italy) for products with ASMR I, II, or III. Although criticised, ERP is still in force (23). How will the health economic evaluation interfere if the prices of such products are set by ERP, based on the ‘accord cadre of 2012’ (24)?
- No information specifying how the CEESP opinion will impact pricing negotiation is available, especially as it may not impact the price for the first 5 years, which is set by ERP (24).
- Recently, the chairman of the CEPS expressed that the CEESP cost-effectiveness opinion is not used for setting listed prices but rather to set the rebates and ensure that the net price is cost-effective. This is inconsistent with the lack of ICER threshold considered, which is an important feature of the French paradigm. Moreover, without any threshold, it becomes extremely difficult to have a transparent discussion on the price.
- At the same time, the chair of the CEPS communicated that rebates above 20% would not be accepted any longer and should lead to price decreases rather than high rebates. If a threshold is available and the listed price is set by ERP and the net price by the ICER threshold, a maximum of a 20% price rebate should be sufficient to render all products cost-effective. Based on the UK's experience, however, this is quite unlikely, as rebates of 50% and more are quite frequent.

### Interactions of CEESP and CEPS

In order to secure a proper interpretation of the cost-effectiveness opinion by the CEPS, the head of the SEESP is often invited to attend the CEPS meetings to provide additional explanations and clarification, resulting in confusion regarding roles and responsibilities. The pricing and reimbursement decision making should be based on the three usual phases: the assessment, the appraisal, and the pricing and reimbursement decision. The assessment gathers exhaustive information and analyses it in an objective way through a predefined framework. In the appraisal phase, this evidence is considered and weighted according to a value judgement. Within the CEESP, this deliberative process is managed within the same committee as the assessment, unlike the UK HTA, National Institute for Health and Care Excellence (NICE). The transparency and reproducibility of this process are only possible if driven by a clear decision framework, which does not exist within the CEESP. Currently, the available decision framework only allows one to address the conformity to the HAS health economics’ guidelines. The lack of standardized appraisal process coupled with the limited insight of the CEPS in health economics explain the need for the CEPS to directly interact with the SEESP head in the decision meetings, which may create confusion and biased decisions. Some of the CEPS members should be qualified to interpret the health economic data properly. The review of the modelling outcome and sensitivity analysis on drug price, performed by the CEESP, should stand alone in a public report to ensure equitable handling of all applicant dossiers. Clear appraisal guidelines should be developed.

### Conditional pricing may become the rule

Although it is not stated in any document, the CEPS likely may be tempted to use real-life studies as a requirement for price re-evaluation. It is recommended by the CEESP to confirm in real life the ICER that is estimated from modelling. This would establish ‘Coverage with Evidence Development’ with or without escrow agreement as a principal in France. France is already quasi-systematically requesting observational drug utilisation studies, often with no specific objectives. In that case, documenting ICER in real life may become the real unreachable challenge for the pharmaceutical industry, although there is a trend that such studies are less requested and/or targeted.

## Perspective

### Preamble

This section stands on the personal opinion of the authors based on their historical experience and insight regarding the French administration processes, their personal interpretation of decisions, and the decision makers’ public presentations and comments. It should be considered speculative, but it has been discussed with experts in the field, including former decision makers who considered it relevant and very plausible. It was neither presented nor discussed with any decision maker on duty.

The introduction of the economic evaluation of health products in France happens within a conflictual environment and important resistance from both the industry and the CEPS members. There have been incisive queries from Parliament and the French Court of Auditors on the lack of use of health economic evidence in price setting in France (3). It was suggested that this was leading to unjustified high prices of pharmaceuticals. Due to substantial resistance, the current regulation is a compromise that is intended to evolve. Therefore, the current process is likely a transition step toward a wider use of health economic evidence.

### Organisation of the CEESP favours the economic approach over public health

Current organization of the CEESP reflects a strong empowerment of health economists in the committee. The CEESP seems much more focussed on economic evaluation than public health, although both are interrelated. Indeed, the CEESP initially created a subcommittee on economics in which only economists participated. Then, as there was no subcommittee for the public health mandate of the CEESP, a subcommittee focussing on human and social sciences was created to balance the roles of the CEESP, thus leaving a narrow place for public health. One vice president used to be a public health specialist, but there are no longer public health specialists in the role of vice president. Finally, the internal assessors are mostly econometricians and very few public health specialists. The public health specialists hired in that position tend to move over time to other positions within or outside the HAS. This imbalance is also obvious when reading the CEESP guidelines and opinions. The CEESP developed a clear decision framework for the economic evaluation, but none is available for the public health assessment. This statement is also in line with the content of the CEESP opinions published to date, in which the technology reviews focussed mainly on economics evidence.

There is a growing trend restricting the public health role of the CEESP to the economics perspective, thus leaving the core scope of public health unaddressed. This seems to be more related to the high expectation and scrutiny of policy decision makers, the Parliament, and the ministry of health on the health economics evidence expected to resolve the budgetary constraint than to a conscious decision to neglect the public health aspect of decisions. It is likely that this situation will not be sustainable in the long run. Decisions should first be driven by the public health interest of the society. Public health could not be restricted to the CT clinical perspective and the CEESP economics perspective. Decisions should integrate the national public health priorities and the public health impact assessed through a robust and transparent decision analysis framework. However, it remains unlikely to be addressed in the short term.

### Extension of health economic evaluation

- In the near future, the question of economic evaluation of products granted an ASMR IV is likely, as such drugs may have a considerable economic impact, despite bringing a modest clinical benefit from the CT perspective. The same question applies to products with ASMR V, but the French HTA and pricing process are already changing rather quickly and ASMR V–granted products may not be a priority. Assessing the ASMR V drugs will also imply important increased resources for SEESP, as most products are granted ASMR V. Moreover, many of the drugs with ASMR IV benefit from an early consultation with the SEESP and file a dossier that is reviewed. The extension of health economic assessment to ASMR IV products may be an important point of discussion in Parliament during the last quarter of 2015 for the Social Security financing bill of 2016. In its report published in 2014, the General Inspectorate of Social Affairs (Inspection Générale des Affaires Sociales, or IGAS) has also pointed out the need to broaden the health economic assessment to a larger number of products, regardless of the nature of the health intervention concerned, notably for clinical practice recommendations or the definition of health strategies (25).

### Merging of the CEESP and the CT

The modelling exercise aims at complementing the clinical evidence as well as providing additional information considered as unavoidable for appropriate decision making (26). There is a duplication of work within the same organisation, HAS (i.e., in the CEESP and the CT), that is unique among all HTA organisations in the world. This duplication may be considered inefficient in future and lead to merging both committees and setting a new harmonised decision framework. Nevertheless, the administration is quite resistant to changes. The principle driving the CT assessment has not changed since its first implementation by the first chairman of the CT. The various attempts to change those rules came to failure (4) even when supported by both the chairman of the HAS and the College (17). The administration and those overseeing health insurance remain very apprehensive as they fear that any change may later affect price, reimbursement, and budgets. However, under increasing budget constraints, accepting duplicate work will soon become difficult, especially when this duplication occurs within the same agency.

It should be noted that medical practice in France is historically primarily driven by clinician research and their experience, rather than by public health research. The role of public health research in France is considerably lower than in Anglo-Saxon countries. In order to be qualified to specialise in public health in France, one must follow the medical doctor education first and then specialise in public health (this is by far the most prominent path). For a long time, this speciality has been a default choice for physicians. Consequently, most decision making and experts’ positions in a broad range of policy-making bodies in France are held by clinicians who tend to favour clinical expertise in decision making for population benefit analysis. A substantial number of Parliament members are also healthcare professionals with clinical experience. Thus, the clinical practice lobby remains important in France and may weigh heavily to maintain a split between the clinical evaluation by the CT and the economic one within the CEESP. In that case, the influence of the CT will decrease, and the ASMR and SMR will become two well-established scores among others generated by the CEESP. The CEPS will be responsible for aggregating the information from both sources in a deliberative process in which ICER will become increasingly important information. In all cases, a change in the balance of power is unavoidable.

It would therefore be expected that the CT holds a qualitative and clinically oriented assessment of efficacy and effectiveness. Simultaneously, the CEESP would hold a comprehensive and quantitative assessment, including relative effectiveness, cost-effectiveness, budget impact, and public health impact, and may become the leading organisation for health intervention assessment for public health decision makers as well as for price and reimbursement setting. However, to ensure that this happens, the CEESP must establish and validate a scoring system that should go beyond ICER to be recognized in France; nonetheless, ICER clearly will become an important and even predominant score among others.

That situation will establish clear-cut responsibilities between consultative technical commissions (e.g., CEESP and CT) that provide a technical opinion and a decision body (i.e., CEPS) that will integrate all information in a deliberative appraisal process to make a policy decision. This will not address the lack of public health focus of both committees but will rather enhance the dual focus on clinical aspects on one side and economic aspects on the other side.

### Establishment of a French threshold

The lack of threshold stands as a real issue in using ICER to inform decision making. This encourages interminable discussion regarding price setting and the lack of a transparency process. Manufacturers often complain about the lack of predictability of price setting in France. Even if a health economic evaluation provided by a manufacturer is rated as fully compliant with the HAS guidelines, this does not indicate how much the payer should compensate for that intervention, although this was the question raised and supposed to be addressed by the legislator through this law.

The lack of expertise to appreciate such information could prove critical for the CEPS. The confusion of mission and responsibility between both committees is a real problem and leads to the question of how much the CEESP/SEESP impacts decisions through potential judgement conveyed to the CEPS. The CEESP is expected to give its opinion to the CEPS (which acts as the decision maker), not to enter into a dialogue. In future, experts in the field may be appointed in the CEPS or as experts to advise the CEPS. It would make sense that current members of the CEESP may become members of the CEPS to help optimise the use of the CEESP's opinion.

There is no doubt about the high integrity of CEPS and SEESP/CEESP members. The issue is compliance with the process so that HTA reports submitted to the payers are transparent enough to stand alone. This does not prevent payers from raising written questions or clarifications to the HTA body.

It is, however, interesting to notice that if the price of products granted ASMR I, II, or III is set by ERP, the discount seems to be set based on the cost-effectiveness. If no threshold is available, the CEESP informs the committee about a range of a pharmaceutical's prices and their related ICER. It is thought that an intuitive moving threshold does exist and is used by the CEPS. This threshold depends on various criteria such as the severity of the condition, the prevalence, the budget impact, the clinical benefit, the availability of alternatives, and the impact on healthcare organisations. As the economic evaluation impacts the rebates, and the rebates in France are composite – even if widely driven by a complex price–volume agreement – and confidential, it is impossible for an outside observer to apprehend this moving threshold. The Chairman of the CEPS acknowledges that his decision of limiting the difference between net and listed price to 20% may not be applicable when setting the rebate with the support of health economic assessment.

The French threshold exists intuitively but not as a hard value. Furthermore, it is not a key driver, as in the UK, but is instead modulated by a number of attributes, which not all are fully explicit. The French threshold ranges from €50,000 per QALY to as high as €300,000 per QALY for some rare conditions or oncology drugs. There is a clear perception that the French informal moving threshold may be outstandingly high compared to that of other countries. For example, in the UK, the threshold is considered to be between £20,000 and £30,000 per QALY. In a recent report, Claxton and colleagues estimated the actual cost per QALY for the UK National Health Service (NHS) at around £13,000 (27). However, more time and decisions are needed to have a clear understanding of the actual French threshold. In France, the decision mainly remains a deliberative decision where the chairman of the CEPS retains a major role through the head-to-head negotiation with the general manager representing the manufacturer. In a recent report from IGAS, a reimbursement decision based on ICER was considered utopian (25). However, it was acknowledged that this is already used for infrastructure building where the life-year is valued at about €50,000. Additionally, the HAS just finalized a review on ICER threshold giving support to the belief that this question is becoming a hot topic (28). Within this report, they referred to the report from the ‘Commissariat general a la stratégie et à la prospective’, which valued a life-year at about €100,000. This review may inform the Minister of Health and the Parliament on the 2016 Social Security financial law to be voted on in late 2015. This would represent a major shift in HTA in France.

### Budget impact analysis to become mandatory

The recent case of sofosbuvir, a new antiviral therapy for hepatitis C virus (HCV) infection, is very illustrative of the limitations of ICER information to address budget constraints. ICER will not address an item's affordability, unless there are appropriate tools to adjust the threshold to horizon-scanning spending-forecast information, or interventions that are unlikely to be fully identified at the time of decision making are displaced.

Following sofosbuvir's market entry, the sustainability of National Health Insurance in many EU countries was threatened because of the drug's potential budget impact. The French administration took a leading role on the executive and legislative sides in the EU. The current price regulation was unable to contain the budget impact of sofosbuvir, and Parliament decided on a yearly budget cap for the HCV anti-infective drugs. This illustrates, on one hand, the inappropriateness of current pricing regulation to control budgets and, on the other hand, the increasing importance of budget impact analysis. It is also interesting to notice that the French silo view prevailed, as the budget cap is drug specific and excludes all other interventions and other costs. Many new innovative therapies that may dramatically change some chronic disease management are expected during the coming decade. If budget impact becomes a critical tool to inform budget cap decision making, it will likely become mandatory together with cost-effectiveness analysis.

### Multi-criteria decision analysis

Although the implementation of a threshold is likely in France, more solutions to enhance the transparency of the appraisal decision framework are under review. Multi-criteria decision analysis (MCDA) is currently under close investigation at the HAS as an alternative to feed the deliberative process. HTA decision making is complex, and multiple facets participate in the evaluation of an intervention. Increasingly, new interventions are competing within the same population with heterogeneous profiles, thus making the decision complex. In the absence of a structured decision process, this leads to inconsistencies among decisions and oversimplification of the questions. MCDA aims to identify and explicate all criteria used in decision making and to provide the relative weight of all criteria in comparison with one another. Then, it should allow defining an aggregated score and a threshold for accepting a new intervention. It is unlikely that such a process will land in France because of methodological issues that remain to be addressed. Nonetheless, being able to set a list of relevant criteria for decision making and grouping them in three classes according to their importance in decision making will already be mazjor steps forward. This will enhance transparency for the appraisal.

### CEESP will broadly inform CEPS

Today, the CT is not required to take drug price into consideration, and the CEESP is, even though it should not be involved in price negotiation. However, when submitting the HTA dossier to the CT, it is expected that the company submits it at the same time as the CEPS pricing dossier. This tends to integrate the price of drugs in decision making, especially for the CT, which is not expected to use this information. It was considered that this may create unwanted interaction between committees that are not expected to exchange prices. Historically, pricing dossiers were presented and discussed with the CEPS after the final conclusion of the CT. In the future, in order to make a distinction between the two steps, this may again be separated and an application dossier filled out sequentially.

The CEESP has a good idea of the manufacturer's expected price as it is critical information in the health economic assessment. If the pricing dossier is submitted sequentially to CEPS, it will grant the manufacturer an opportunity to integrate the HTA decision in the pricing dossier. It is logical to use the outcome of HTA opinion to develop the pricing dossier or define the expected price. This is also a change that is likely to be considered by Parliament in the near future.

For pricing benchmarks, the CEPS uses the daily price, episode-of-care price, or yearly price of the reference product. These discussions occur within the CEPS without involvement of the HTA bodies, and as such, they remain independent of all public health, epidemiologic, or clinical expert considerations. They are primarily driven by an accounting perspective. As most drugs are expected to be granted an ASMR IV or V in France, they are expected to be priced at a level that implies no budget impact on the therapeutic class. However, the way a drug may be used on a specific target population (at a specific dosage or duration) compared to currently available drugs on the market may more or less have a legitimate impact on the therapeutic class budget. The CEPS mission and expertise are not primarily in epidemiology and public health and forecasting models, despite the fact that eminent experts are members of the CEPS. In future, these studies, performed by the manufacturer and deeply scrutinised and discussed within the CEPS, may be reviewed and amended by the CEESP, as they are within its core expertise.

### CEESP will review and inform early-entry agreements

Finally, the CEPS systematically develops a number of confidential contractual agreements associated with the market access of new drugs called under a contract-called convention. This may include price–volume agreements (the most common in France), coverage with evidence development, payment for performance, and eventually risk-sharing agreements and various forms of rebates, discounts, linkage to benchmark drug price, and so on. As in the UK, where the HTA NICE assesses the impact of such agreements for the Department of Health and the NHS, it may become the CEESP's mission to review those agreements in the future, to model the actual net price under various assumptions, inform the CEPS decision making. This would seem justifiable as they have the expertise to execute that mission and ensure a more accurate budget impact analysis of such market access agreements.

## Conclusion

Although requested by various high-level bodies in France, the Manufacturers’ Union as well as the CEPS delayed the introduction of health economic assessment in new technology price setting. The situation did change gradually, but over a period of 8 years, it has been a dramatic transformation. The legislator and executive administration may have not fully anticipated the areas of inefficiencies, conflict, and confusion associated with this law. However, it may have also resulted from political compromises. The issued application decree still leaves some areas of uncertainty that must be addressed surrounding the introduction of health economics in market access of drugs in France. The decree creates confusion and conflicting information with respect to existing pricing regulation and practice, especially for the ERP. The experience will set the new practice and rules. However, active discussions are still ongoing in France on the development of the use of health economics to inform public health decision making and ensure the optimal use of available resources to maximise the population's health. At present, the major issue relates to the lack of an ICER threshold, which seems circumvented by the CEPS and the CEESP. The current situation should be considered a transitional period, and major steps are expected to occur in the near future. The 4-year cycle may continue to be the appropriate tempo for future reforms: 2016 (as did 2012 and 2008) will fall 1 year before presidential and Parliament elections, which is appropriate timing for impactful reforms. The application decree will arrive after the election, which will leave a peaceful window before actual implementation, as for the 2012 law (2). This is an opportunity to show a strong will to reform with no risk of overreaction. Empowerment of the CEESP (merged or not with the CT) is expected, and it may become the unique or leading committee addressing the HTA of pharmaceuticals in France. However, it is likely that the robust and well-established methodology developed by the CT to assess comparative efficacy or effectiveness will remain in force. The reinforcement of a standardized appreciation of public health impact of new technologies may remain neglected as successive French administrations have shown historically and currently very little appetite for this field. The development of a transparent appraisal decision framework will likely emerge.
